# Food allergy knowledge, attitudes and beliefs: Focus groups of parents, physicians and the general public

**DOI:** 10.1186/1471-2431-8-36

**Published:** 2008-09-19

**Authors:** Ruchi S Gupta, Jennifer S Kim, Julia A Barnathan, Laura B Amsden, Lakshmi S Tummala, Jane L Holl

**Affiliations:** 1 Institute for Healthcare Studies, Northwestern University, Feinberg School of Medicine, Chicago, IL, USA; 2 Department of Pediatrics, Children's Memorial Hospital, Northwestern University, Feinberg School of Medicine, Chicago, IL, USA; 3 Division of Allergy, Children's Memorial Hospital, Chicago, IL, USA

## Abstract

**Background:**

Food allergy prevalence is increasing in US children. Presently, the primary means of preventing potentially fatal reactions are avoidance of allergens, prompt recognition of food allergy reactions, and knowledge about food allergy reaction treatments. Focus groups were held as a preliminary step in the development of validated survey instruments to assess food allergy knowledge, attitudes, and beliefs of parents, physicians, and the general public.

**Methods:**

Eight focus groups were conducted between January and July of 2006 in the Chicago area with parents of children with food allergy (3 groups), physicians (3 groups), and the general public (2 groups). A constant comparative method was used to identify the emerging themes which were then grouped into key domains of food allergy knowledge, attitudes, and beliefs.

**Results:**

Parents of children with food allergy had solid fundamental knowledge but had concerns about primary care physicians' knowledge of food allergy, diagnostic approaches, and treatment practices. The considerable impact of children's food allergies on familial quality of life was articulated. Physicians had good basic knowledge of food allergy but differed in their approach to diagnosis and advice about starting solids and breastfeeding. The general public had wide variation in knowledge about food allergy with many misconceptions of key concepts related to prevalence, definition, and triggers of food allergy.

**Conclusion:**

Appreciable food allergy knowledge gaps exist, especially among physicians and the general public. The quality of life for children with food allergy and their families is significantly affected.

## Background

Food is ubiquitous in our society and is an important feature in social activities, particularly for children. Food allergy has recently been increasing in prevalence [1,2]. In the United States, food allergy affects up to 4% of the adult population and 6–8% of children [3-5]. Ninety percent of schools enroll more than one child with a food allergy, and half of these schools have had a child experience an allergic reaction in the past two years [6].

Food allergy is defined as an adverse immune response to specific foods, typically proteins [3]. In children, there are eight foods that cause 90% of food allergy [7]. The diagnosis is based on clinical history and can be supported by testing, such as skin prick testing, specific IgE and oral food challenges. IgE-mediated food allergy can lead to anaphylaxis [8] and anaphylaxis has been shown to recur in 93% of food-allergic children who experience an anaphylactic reaction [9]. It is estimated that 150 Americans die each year due to food allergy [10], with most death occurring among adolescents and young adults [11]. Currently, management of food allergy primarily consists of strict avoidance of the offending food allergen and the initiation of therapy in case of an ingestion [4,6,12].

Organizations like the Food Allergy and Anaphylaxis Network (FAAN) and local parent support groups have been advocating for increased knowledge and awareness of food allergy nationally and at the community level [13,14]. However, previous research shows that misconceptions around food allergy prevalence exist among the general public [4,15], and that physicians' knowledge of food-induced anaphylaxis is lacking [16].

Moreover, it has been well-established that families of children with food allergy have a lower quality of life [17-21]. Food allergy has been shown to lower general health perception, limit family activities, and have a significant emotional as well as economic impact on the parent [17,18]. Delayed diagnosis by physicians and social stigmatization by the general public may be factors leading to further difficulties which parents of children with food allergy face in dealing with the daily fear of a life-threatening reaction.

Thus with the increasing prevalence of food allergy and the absence of a cure, community awareness about the signs and treatments of allergic reactions is vital. The purpose of this study, therefore, is to better understand the current knowledge, attitudes, and beliefs surrounding food allergy among three groups: (1) parents of children with food allergy, (2) primary care physicians (pediatricians and family physicians), who are often the first to diagnose a food allergy in a child, and (3) the general public. Based on these findings, the final goal of this study is the development of three validated survey instruments to assess knowledge, attitudes, and beliefs of parents, physicians, and the general public.

## Methods

### Participants

Three populations were recruited to participate in the focus groups: parents of children with food allergy, pediatricians and family physicians, and the general public. All focus groups took place between January and July of 2006 in the Chicago area.

Three focus groups of upper income parents of children with food allergy were conducted. Mothers and fathers participated in separate groups in order to foster a comfortable atmosphere for discussion, particularly regarding issues about family relationships. Participants were recruited through e-mail notices to members of the support group Mothers of Children Having Allergies (MOCHA). One focus group of 4 mothers was conducted in the city of Chicago, and a second group of 9 mothers and a group of 5 fathers were conducted separately in a Chicago suburb.

Three physician focus groups were conducted, two with pediatricians and one with family physicians. Physicians were recruited through clinics and hospitals and through contacts at the American Academy of Family Physicians. Recruitment flyers were disseminated via e-mail, and recruitment phone calls were made to physician practices. One focus group of 6 pediatricians was held in the city of Chicago, but technical difficulties with recording limited the usable data from this focus group. However, the themes and responses from this focus group were consistent with those from the other two physician focus groups. A second group of 4 pediatricians was held in a Chicago suburb. The focus group of 4 family physicians was held in the city of Chicago.

Two general public focus groups were conducted. One group was comprised of 5 lower-income parents with children insured by Medicaid who were recruited through a pediatric clinic. The second general public focus group was comprised of 9 upper-income parents recruited through an elementary school in an affluent Chicago neighborhood.

Locations of focus groups were selected based on convenience for participants, including a school, an office building, a hospital, and a medical clinic. Refreshments were provided and transportation assistance offered. At the conclusion of the focus groups, participants received a $25 gift card.

### Creation of content domains and focus group questions

Focus groups were held as a preliminary step in the development of validated survey instruments to assess food allergy knowledge, attitudes, and beliefs of parents, physicians, and the general public on a national scale. The methodological framework was based upon the development of an instrument with like objectives used to assess knowledge, attitudes, and beliefs of asthma [22]. Development began with a review of previous food allergy literature to better understand important knowledge areas as well as current food allergy attitudes and beliefs. Informal discussions were also held with local parents of children with food allergy, physicians, and the general public. Based on review of current literature and expert opinion, relevant content areas of food allergy were established.

An expert panel of 9 individuals was assembled, comprised of community pediatricians, two pediatric allergists with expertise in food allergy, survey researchers, a leader of the largest US food allergy advocacy network, and a parent of two children with food allergy who also founded the first local support group. Based on the panel's review, 8 final content domains were identified: (1) definition and diagnosis, (2) symptoms and severity, (3) triggers and environmental risk, (4) perceptions of susceptibility and prevalence, (5) stigma and acceptability, (6) perceptions of quality of life, (7) treatment and utilization of healthcare, and (8) policy issues. These domains formed the framework from which questions were developed for the focus group protocol, a standardized set of open-ended questions, with minor variations based upon the group type. Examples of the questions and study domains are listed in Table 1.

**Table 1:** Sample focus group questions by domain.

Domain	Parent Focus Groups	Physician Focus Groups	General Public Focus Groups
Definition & Diagnosis	▪ In your own words, tell us what a food is.	▪ How do you define anaphylaxis?	▪ What does it mean to you when you hear someone is allergic to a food?
	▪ How is a food allergy diagnosed?	▪ How do you make a diagnosis of food allergy? What tests do you use?	▪ How does a doctor decide a child has a food allergy?

Symptoms & Severity	▪ What are the signs that make you think your child has a food allergy?	▪ What signs and symptoms alert you to a possible diagnosis of food allergy?	▪ What happens when someone has an allergic reaction to a food?
	▪ How sick can a child become when they are having an allergic reaction?	▪ Does anaphylaxis require respiratory, laryngeal, or gastrointestinal symptoms?	▪ How sick can someone with a food allergy can be? Could he/she die?

Triggers & Environmental Risk	▪ What do you think causes food allergy (environmental factors, genetics, dietary factors)?	▪ What are the risk factors for development of food allergy (environmental factors, genetics, dietary factors)?	▪ Can your child catch a food allergy?
	▪ Do you think what the mother ate during her pregnancy makes a difference in whether a child has a food allergy?	▪ What are some co-morbidities associated with food allergy?	▪ Do food allergies run in families?

Perceptions of Susceptibility & Prevalence	▪ How common is food allergy?	▪ To what foods are children most commonly allergic?	▪ In a group of 100 children, about how many of them do you think would have a food allergy?
	▪ To what foods are children most commonly allergic?	▪ Does the prevalence of food allergy vary by race, ethnicity, or socioeconomic status?	▪ To what foods are people most often allergic?

Stigma & Acceptability	▪ Do you feel like you have to explain your child's allergy to friends and family? How well do they accept this?	▪ What kinds of difficulties might children with food allergy and their parents face in school, at home, between parent and child, etc.?	▪ What would you think if your friend told you that his/her child had a food allergy?

Perceptions of Quality of Life	▪ How does having a child with food allergy affect your family and social life?	▪ What are some personal and social issues for the children and families with food allergy?	▪ How hard do you think it is to care for a child with food allergy?

Treatment & Utilization of Healthcare	▪ What are the main ways food allergies are treated?	▪ How can food allergy be prevented?	▪ Do you think food allergy can be treated so that it will go away?
	▪ How difficult is it for you to follow the doctor's instructions?	▪ What are the key treatments for food allergy?	▪ How do you treat people with food allergy?

Policy Issues	▪ What public policies would help families with children with a food allergy?	▪ What public policies would help families with children with a food allergy?	▪ What laws could help families of children with food allergy?

### Conducting the focus groups

Investigators experienced and trained in leading focus groups guided the discussions. Standard moderation techniques were used throughout [23]. Focus group participants received equal time for responding to questions. All focus groups lasted 1–2 hours and were audiotaped and transcribed. Focus groups continued until all discussions on pertinent topics were exhausted.

Focus group participants were informed that the discussion would be recorded. Each participant verbally agreed to participate. Participants were addressed by a color (e.g., Ms. Pink, Dr. Blue, Mr. Green) during the discussion. No identifiable information was collected during the focus group sessions. After an introduction and explanation of the focus group format, the protocol questions were posed to each group.

### Analysis

The focus group audiotapes were transcribed by independent medical transcriptionists. Upon review of initial transcripts, a coding scheme was developed within the framework of the eight content domains. A constant comparative method was used to identify emerging themes [24,25]. Codes were modified and expanded based upon review of subsequent focus group transcriptions. Coding was facilitated by a qualitative data analysis software program, *Atlas.ti*. At least two reviewers independently coded each transcript, followed by reconciliation of the codes to produce a single coded transcript. Discrepancies were reconciled based on the transcript language and code definitions until complete agreement of the assigned code was met.

The study was approved by the Children's Memorial Hospital Institutional Review Board and the Northwestern University Feinberg School of Medicine Institutional Review Board.

## Results

### Parents of children with food allergy

Parents had children with food allergy ranging in age from 1–14 years with half being male. The age at which a food allergy was diagnosed ranged from 6 weeks to 8 years, with a median age of 1.25 years. All food allergy diagnoses were made by a physician and all children had positive clinical symptoms consistent with IgE-mediated food allergy. Parents had children allergic to the following foods: milk, egg, soy, peanut, tree nuts, fish, shellfish, sesame seed, potato, legumes, and beef. Children of participants had severe food allergy: per self report, about two-thirds of participants' children had experienced anaphylaxis. Parents also reported that about one-third of the children had developed tolerance to at least one food and that more than 75% were allergic to multiple foods.

The dominant theme in both the mother and father focus groups was the effect on quality of life (Table 2). Parents reported that the life-threatening nature of food allergy evoked strong emotions of fear, guilt, and even paranoia. Parents experienced significant anxiety about keeping their children safe from allergenic foods and expressed concern regarding the possible negative repercussions of parental hypervigilance on their children. Challenges associated with daily living such as attending school, going to restaurants, and visiting friends, had a major impact on parental quality of life. Both mothers and fathers agreed that food allergy affected decision-making in daily social activities.

**Table 2:** Parent focus groups: quotations from mothers (M) and fathers (F) of children with food allergy arranged by domain.

Domain: Perceptions of Quality of Life	
Emotional Response	▪ (M): "One of my sons has had two anaphylactic reactions, and it never leaves you. You cannot ever forget. It's like a broken record.....just the feverishness, and this helplessness, horrible helplessness feeling you cannot describe. It is such a gut-wrenching squeezing of your body when you see your child going through that."
	▪ (F): "You are so fearful of anaphylaxis and death. It is this kind of unknowing, this uncertainty of what degree of reaction it's going to be, and this constant [feeling]."

Impact on Relationships	▪ (M): "You do find that [in] every relationship you have to address the allergy issues. So you are always talking about it because there is always something that can come up."
	▪ (F): "I would say from my family's standpoint it was very difficult at first for them to understand, and in fact their tendency was to say, 'Well, you are just being neurotic and overprotective, and it is really not an issue'. And frankly, it was almost that they did not want to be inconvenienced by our situation. Therefore it was easy for them to discount it and say that [we as] the parents [were] being overprotective."

Effect on Daily Social Life	▪ (M): " [Food allergy] affects every aspect [of your life] from where you take a vacation to whether and where you go out for dinner to play dates, which we certainly cannot do on the spur of the moment sort of basis as many [other] families can do. It affects literally every aspect of our social life."
	▪ (F): "Going to parties, going to restaurants, having people over, any social contact that involves food becomes very distracting. [You] always kind of have to make sure that they [food-allergic child] are not eating anything that they have not asked you about."

Effect on Marriage	▪ (F): "My wife is much more into 100% prevention all the time and I am much more into trying to maximize what my son can do. [I am] trying to figure out what that limit is so he can do the most he could possibly do where [as] she is much more in tune to 'Let's not take a chance at all' and 'I do not want put my son in danger."'
	▪ (F): "I think we experience stress in different ways, but I think the fundamental difference is that she [the child's mother] feels the day to day responsibility [due to] the fact that she is there, and she is making those decisions. [Food allergy] is [the] one area in our marriage that [arguments] come up because I do not think I am as aware of the amount of stress that it creates for her."
	▪ (M): "Milk and cheesy things are favorite [s] of [my husband's], and he is not going to give it up. [He is] not going to give it up regardless if it is life threatening to our child. So [milk] is in the house, and I follow him around and clean up. It has caused a huge marital strain."

Domain: Definition & Diagnosis	

Diagnosis	▪ (M): "It literally took six physicians before one said, 'Let's see if maybe this [eczema] is food-related with your daughter.' [There were] six [physicians] before someone thought that [food allergy] may be a possibility, and then when we went back to our dermatologist, we were basically told that it was just a lucky coincidence that when the dairy was [removed from her diet], the eczema went away too. I do not think that the doctor was being ignorant. I think that perhaps the doctor just did not know that this was even a possibility because of her training."
	▪ (M): "I think there was a time [when] food allergies were a rare thing, and I think pediatricians did not have the same level of concerns for it, just [like when] childhood diabetes was a rare thing. I think there is a bit of a lag, and a need for the medical community, especially pediatricians, to step up in terms of their level of understanding of the prevalence of this illness."

Parents also described emotional tension in family relationships due to lack of understanding by extended family members that a food may be life-threatening to the child or that there may be hidden allergic or cross-contaminating ingredients. Marital tensions and conflict were prominent due to differences in parenting philosophy and practice. Mothers and fathers had differing opinions regarding the measures needed to protect the child from allergic reactions. Mothers tended to shelter their children from the smallest possibility of risk whereas fathers expressed a desire to expand their child's life experiences as much as possible.

Finally, parents reported frustration and difficulties in receiving a timely diagnosis and recommendations with regard to management of food allergy. Parents felt that insufficient physician knowledge of the disease was problematic. Parents also felt that physicians of different specialties (e.g., pediatrics, dermatology, allergy/immunology, etc.) provided conflicting guidance in the diagnosis and treatment of food allergy.

### Pediatricians and family physicians

Physicians surveyed had been in practice from 5–28 years. The family physicians had minimal experience with food allergic children while the pediatricians saw 1–2 cases per month on average.

Physicians were able to identify the respiratory and cardiovascular symptoms associated with anaphylaxis (Table 3). Both pediatricians and family physicians accurately named milk, egg, and peanut as the most common allergens in children. Some physicians reported that strawberries and tomatoes were foods that commonly cause allergic symptoms. Both family physicians and pediatricians demonstrated a lack of knowledge about food allergens to which children are likely to develop tolerance, specifically in IgE-mediated food allergy. The discussion of food allergy generally led the physicians to the diagnosis of 'milk protein allergy,' and not all physicians immediately differentiated benign proctocolitis (blood in stool) from IgE-mediated food allergy. Physicians expressed a wide range of opinions around their recommendations for breastfeeding and solid food introduction and the relationship of these factors with the development of food allergy.

**Table 3:** Physician focus groups: quotations from pediatricians (Ped) and family practitioners (FP) arranged by domain.

**Domain: Definition & Diagnosis**	
***Definition***	

*Anaphylaxis*	▪ (Ped): "A sudden distressing feeling that is difficulty breathing, possibly associated with swollen tongue and rapid heart rate [and] drop in blood pressure."
	▪ (FP): "A life threatening reaction to some offending agent..."
	▪ (FP): "including impending circulatory collapse"

*Common Allergens*	▪ (FP): "Peanuts...like nuts. What comes after that, I guess milk...wheat."
	▪ (FP): "Fish. Strawberries are fairly common too I think."
	▪ (Ped): "...shellfish, eggs, wheat, soy."
	▪ (Ped): "Tomatoes."

*Prognosis & Development of Tolerance*	▪ (Ped): "I really do not know.... I have no concept of what percent outgrow [food allergy]"
	▪ (FP): "One would think that [food allergies] are [more] prevalent the older you get because you [would] have more chance [s] to develop more allergies."
	▪ (Ped): "So far all my milk protein allergy victims that have reached the age of two today have in fact outgrown the milk protein allergy."

*Breastfeeding & Solid Food Introduction*	▪ (Ped): "Generally [if] there is a family history of peanut allergy, I suggest they wait [to introduce peanut] until three [years]. Then if there is no family history, [I suggest they wait] until two [years]."
	▪ (FP): "I do not think I have ever specifically given anticipatory guidance about [when to introduce] peanuts."
	▪ *Interviewer: Do you have any recommendations that you give to breastfeeding mothers? *(Ped): "Nothing specific." (FP): "I do not give a particular diet instruction other than to avoid caffeine."
	▪ *Interviewer: What are the risk factors for the development of food allergy? *(FP): "I wonder if breastfeeding [would] decrease [the] risk of developing food allergies, but maybe women who breastfeed attempt to delay introduction of solids to the baby. So I am not sure." (Ped): "If you start [solids] earlier there is an increased risk of allergy because your baby's gut is more permeable, and those substances will go through and can stimulate their immune system. So it is better ▪ to delay."

***Diagnosis***	

*Diagnostic Criteria*	▪ (Ped): "I do not ever treat eczema as food-related. It may be. It may not be. I never took it serious [ly] enough to react that way. Treat it [with] a little basic hydrocortisone or emollients. It gets better and move on."
	▪ (FP): "I actually kind of find out about a food allergy at the back end of an investigation for maybe persistent asthma or a persistent dermatological condition [rather] than discovering [food allergy] by testing. That has been my experience."

*Reliability of Clinical History*	▪ (Ped): "I have seen some fish allergies, and [allergies to] tree nuts. I am not sure how seriously to take them. I have one family that believes that their child has a citrus allergy despite the fact that I have told them it is probably just a response to the acid in the citrus, but they have self-diagnosed [this allergy]."
	▪ (FP): " [When making a diagnosis, I rely on] a parent telling me that there is some kind of skin breakout after eating something."

The physician focus groups acknowledged that skin prick testing and *in vitro *blood tests for specific IgE were methods that could be used in the diagnosis of food allergy. They also agreed that a trial elimination of a suspected allergen could lead to a diagnosis. However, the physicians had differing opinions regarding whether the presence of eczema or asthma suggested the possibility of food allergy. Several also stated that they were unable to obtain a sufficient history to make a diagnosis.

### General public

Twelve of the 14 general public participants had children under the age of 18. The general public focus groups had many misconceptions around the definition and symptoms of food allergy (Table 4). The level of knowledge and understanding varied widely. General public participants understood that some individuals outgrow their food allergy, yet there were conflicting ideas about when and how this occurs. Some symptoms of food allergy were correctly identified but behavioral conditions, such as attention deficit disorder, were incorrectly attributed to food allergy. In addition, the understanding of susceptibility differed. These groups reported the prevalence of food allergy in the United States to be between 20–100%, higher than current estimates and even higher than overestimates noted in prior publications [4,26]. Many general public focus group participants in this study stated that their children were allergic to fruits without a formal physician diagnosis. When discussing quality of life for families and children with food allergy, the general public described their experiences on airplanes and play dates with food allergic children.

**Table 4:** General public focus groups: quotations from higher (H) and lower (L) income participants arranged by domain.

**Domain: Definition & Diagnosis**	
*Food Allergy*	▪ (H): "I think that [food allergy] may have something to do with the compromised immune system. There are a lot of factors that can affect your immune system that could be hereditary, but [food allergy] could also be due to environmental factors or conditions at birth or early illness or something that leaves the immune system in a weakened state or perhaps in a state where it is malfunctioning."
	▪ (L): " [Food allergy is when] enzymes aggravate the nerves inside [and] stimulates pain to the brain."

*Development of Tolerance*	▪ (H): "My husband was allergic to fish, and his brother was allergic to nuts, and they did not pay any attention to [avoiding] them and they outgrew [their allergies]."
	▪ (L): "If you can find out what the [im]balance is in the system, I believe it could be corrected. I do not know about the shellfish [allergy] though because that is roughest [allergic reaction] I [have] ever seen."

**Domain: Symptoms & Severity**	

*Symptoms*	▪ (H): "I think of it as not just a skin reaction but sometimes [of] a throat closing."
	▪ (L): "I think that [symptoms would include] itching, swelling definitely, [and] even throwing up."
	▪ (H): "I believe that perhaps my son's ADD [attention deficit disorder] is linked to food allergies."
	▪ (L): "I think I am suspecting that I might have an allergy to pineapples. I have never been diagnosed with them, but I get itching and the swelling of the tongue and a really bad headache every time I eat fresh pineapple, but I can drink pineapple juice."

**Domain: Perceptions of Susceptibility and Prevalence**	

*Susceptibility*	▪ (L): "I think that sometimes allergies come through the system like your mother is allergic to something; sometimes the children are allergic to it too."
	▪ (H): "My kids had nuts, strawberries, everything [to eat]. I have no history of food allergies, and neither does my ex-husband. I exposed my kids to whatever they wanted to eat when they were very young, and hence I have two non-picky eaters. They will eat anything, and they are not allergic to anything."

*Prevalence*	▪ *Interviewer: If we had a group of a hundred children in this room, how many children do you think would have a food allergy?*
	(H): "I really think that every single person has an allergy that they do not know about."
	(H): "I was thinking more like 20%."
	(L): "I am going to say 50%."

**Domain: Perceptions of Quality of Life**	

*Public Impact of Food Allergy*	▪ (H): "I used to be in advertising [and] I know that Southwest's slogan is 'Fly for peanuts.' I just cannot believe that [in] this day and age that they [could] be in such a confined area 35,000 feet from any hospital [and] serve peanuts. I think it is crazy."
	▪ (H): "Unless they make a specific announcement, 'We have a highly allergic passenger on board [an airplane],' no one is going to think twice about opening whatever it is they have. So the responsibility lies on the parent or the child as they become older, and [they should] take on the responsibility to let other people know."
	▪ (H): "One of my daughter's friends is allergic to nuts, and I have to be very careful when we have her over for a play date."

## Discussion

Food allergy knowledge, attitudes, and beliefs varied significantly across the three populations represented by our focus groups. In our study, parents of children with food allergy had a solid foundation of food allergy knowledge, diagnosis, and treatment. They emphasized the significant impact that food allergy had on their daily quality of life. Pediatricians and family physicians had familiarity with the definitions of food allergy and anaphylaxis. However, there were inconsistencies in their understanding of symptoms suggestive of a food allergy diagnosis. The general public had significant variations in their knowledge about the definition, symptoms, and prevalence of food allergy.

The most striking aspect of the parent focus group discussions was how managing a child's food allergy had a significant negative impact on personal and familial quality of life. Parents felt that food allergy impacted not only their daily social lives but their relationships with their spouses and extended family. Parents confirmed that food allergy affected their child psychologically and socially, as described in previous studies [27].

A novel finding from this study was the mothers' assertions that their child's food allergy caused them to stop working outside the home. They described the need to remain at home full-time to keep their child safe. It was difficult for many mothers to entrust others with the care of their child. Consistent with previous quality of life studies, parents in our focus groups confirmed that food allergy had a significant impact on their general health perception, their emotional state, and their social activities [17,18]. Mothers also seemed to experience a considerably stronger negative impact on quality of life when compared with fathers.

Prior study has documented the short-comings of medical professionals in the provision of medical information and practical recommendations to parents concerning their child's food allergy [28]. Our study reiterated this concern, emphasizing parents' dissatisfaction with their primary care physicians' ability to diagnose and manage their child's food allergy. Parents felt physician knowledge was poor and inconsistent across specialties.

Physicians had basic knowledge of food allergy and anaphylaxis but differed in their approach to diagnosis and the advice they offered families about breastfeeding and introduction of solids. Existing research confirms that recommendations vary among experts with regard to diagnostic evaluations for food allergy and food-induced anaphylaxis [5,29-32]. High rates of misdiagnosis have also been described in the emergency room setting [33]. There was considerable variation among recommendations given by physicians as reported by our focus groups. For example, parents reported being told by physicians that eczema was not a manifestation of food allergy (Table 2). In fact, approximately 35% of young children with atopic dermatitis have food allergy, particularly in infants and toddlers whose disease is more severe or recalcitrant to therapy [34,35].

Guidelines for diagnosis and management have been published, including practice parameters for food allergy [36] and anaphylaxis [37]. Moreover, delay of solid food introduction until after 4 months of age and the use of extensively or partially hydrolyzed formulas have been recommended as evidence-based guidelines for infants at high risk for development of atopic disease [38,39]. However, delaying introduction of highly allergenic foods, although historically prescribed as a prevention tactic [40], is not evidence-based [38,39]. In spite of the availability of these published guidelines, there is a need for improved continuing medical education among physicians with regard to food allergy.

The general public varied in its knowledge of the definition, symptoms, and triggers of food allergy. They tended to overestimate food allergy prevalence, which is consistent with previous literature [4]. Participants also reported having a food-allergic child absent a formal diagnosis; prior study shows that only approximately 40% of patients' histories of food-induced allergic reactions can be verified [41-43] and that individuals are inclined to overdiagnose food allergies in themselves or in their children [44]. However, participants reporting a fruit allergy absent a formal physician diagnosis may be an exception. These reports may indicate oral allergy syndrome (or pollen-food allergy), which is an IgE-mediated phenomenon, resulting in immediate oral pruritus after ingesting certain fresh fruits or vegetables [45]. These patients typically have a history of seasonal allergic rhinitis (hay fever) as the sensitizing pollens have proteins that are homologous to certain fruits and/or vegetables. Because these proteins are labile to heat, individuals typically react only to fresh forms of these foods. Moreover, systemic reactions are averted as these proteins are easily digested.

The misconceptions among the general public support the perception that the plight of parents of children with food allergy is not well understood. Parents (of children *without *food allergy) will likely come in contact with food-allergic children in their neighborhoods or through schools and camps. It is vital for those in contact with children to have accurate awareness of food allergen avoidance and treatment in order to prevent serious, or even fatal, allergic reactions.

There are limitations to the study design. Selection bias is inherent as participants in the focus groups volunteered themselves and may therefore be more motivated or more knowledgeable than the average person in each targeted population. Particularly, parent participants were selected from a food allergy support organization, and may therefore be more knowledgeable about food allergy and have more severely food-allergic children [46]. The total number of individuals in each focus group was small and based in the Chicago area and therefore not necessarily representative of parents, physicians, or the general public across the US. However, even in this select population, we found a substantial lack of knowledge and a considerable amount of misinformation.

Through these focus groups, we obtained valuable information that will be used in the development of a question bank for surveys assessing the food allergy knowledge, attitudes, and beliefs of parents of children with food allergy, physicians, and the general public. Each survey will be administered nationally in order to assess existing food allergy knowledge gaps. This will allow for interventions targeting key misconceptions in food allergy knowledge for parents, physicians, and the general public. Understanding how food allergy is stigmatized may also help devise methods to improve the quality of life of affected families.

## Conclusion

In summary, these focus groups have been the first step in understanding the true gaps that exist in food allergy knowledge, attitudes, and beliefs among three very distinct populations. Increasing awareness of published practice parameters or dissemination of evidence-based guidelines as well as increasing food allergy curriculum in resident education may help decrease variation in physician practice. Significant efforts are needed to improve the food allergy knowledge of the general public, especially those in contact with children. Until a cure is found or better treatments developed, improving knowledge of symptoms, treatment, and prevention is the best strategy to protect children from potentially fatal reactions. Larger studies are needed to verify key knowledge gaps and misconceptions around food allergy. Interventions aimed specifically at these knowledge gaps may help improve the health and lives of children and families with food allergy.

## Competing interests

The authors declare that they have no competing interests.

## Authors' contributions

RSG developed the initial design of the study, participated in the focus groups, reconciliation of the transcripts & analysis and drafted the manuscript. JSK helped develop the initial design of the study, participated in the focus groups, reconciliation of the transcripts & analysis and edited the manuscript. JAB coordinated the focus groups, reconciled the transcripts, developed the tables and edited the manuscript. LBA coordinated the focus groups, reconciled the transcripts and edited the manuscript. LST helped with the initial design of the study and assisted in the initial drafting and editing of the manuscript. JH helped develop the initial design of the study, participated in the focus groups, reconciliation of the transcripts & analysis and edited the manuscript. All authors read and approved the final manuscript.

## Pre-publication history

The pre-publication history for this paper can be accessed here:


